# A lytic bacteriophage vB_KpnP-6K2 inhibits ST11-KL64 *Klebsiella pneumoniae* induced cell death and inflammatory response

**DOI:** 10.3389/fcimb.2026.1749949

**Published:** 2026-02-20

**Authors:** Zhaoyi Pan, Jing Fan, Xianbo Geng, Shujuan Zhang, Huijiao Zhang, Shujun Liu, Ling Zhang, Guangjian Xue, Rui Li, Tianle Li, Xiaofeng Liu, Yating Yu, Na Wang, Changzhong Jin, Nanping Wu

**Affiliations:** 1 Jinan Microecological Biomedicine Shandong Laboratory, Jinan, China; 2 Shandong First Medical University and Shandong Academy of Medical Sciences, Jinan, China; 3 State Key Laboratory for Diagnosis and Treatment of Infectious Diseases, National Clinical Research Center for Infectious Diseases, Collaborative Innovation Center for Diagnosis and Treatment of Infectious Diseases, The First Affiliated Hospital, Zhejiang University School of Medicine, Hangzhou, China

**Keywords:** bacteriophage, bloodstream infections, cell death, inflammatory response, *Klebsiella pneumoniae*

## Abstract

**Introduction:**

The global dissemination of multidrug-resistant *Klebsiella pneumoniae* (*Kpn*) underscores the critical demand for alternative therapeutics such as bacteriophages. This study characterizes a novel bacteriophage, vB_KpnP-6K2 (6K2), isolated against a clinically relevant ST11-KL64 *Kpn* strain, and evaluates its potential for therapeutic application.

**Methods:**

Phage 6K2 was morphologically examined by transmission electron microscopy and genomically analyzed via whole-genome sequencing. Its stability across pH and temperature ranges, adsorption kinetics, and burst size were determined *in vitro*. The inflammatory response to Kpn infection was assessed in HEK293T, A549, Hela, and THP-1 monocytic cells by measuring cytokine and chemokine expression, while cell death was evaluated in A549 lung epithelial cells. The therapeutic efficacy of 6K2 was tested in a lethal murine systemic infection model, where a single intraperitoneal dose was administered one-hour post-bacterial challenge. Survival, bacterial clearance, and phage kinetics in blood were monitored.

**Results:**

Phage 6K2 exhibits a polyhedral head and short tail, classifying it within the Podoviridae family (Autographiviridae family, *Przondovirus* genus). Its double-stranded DNA genome comprises 40,147 bp. The phage demonstrated stability across a broad pH (4-12) and temperature (4-50°C) range, rapid adsorption, and a burst size of 13.6 PFU/cell. *In vitro*, *Kpn* infection significantly upregulated inflammatory mediators in THP-1 cells and induced death in A549 cells; both responses were potently inhibited by 6K2 treatment. In the murine infection model, a single dose of 6K2 achieved 100% survival, accompanied by rapid clearance of bacteremia and high initial phage titers in the blood.

**Discussion:**

These findings highlight the promising potential of bacteriophage 6K2 as an effective therapeutic agent against multidrug-resistant *Kpn* infections. The phage not only suppresses bacterial load but also mitigates infection-associated inflammatory responses and cellular damage. The complete rescue in a lethal systemic infection model underscores it’s *in vivo* efficacy and supports further development of phage-based strategies for combating resistant bacterial infections.

## Introduction

*Klebsiella pneumoniae* (*Kpn*) represents a predominant cause of antimicrobial-resistant opportunistic infections among hospitalized patients, owing to its remarkable capacity to acquire exogenous genetic elements encoding both resistance and hypervirulence (Wyres et al., 2020; Yang X. et al., 2021). As a key contributor to the global antibiotic resistance crisis, *Kpn* is implicated in more than 100,000 deaths annually worldwide. Strains resistant to carbapenems and third-generation cephalosporins represent particularly urgent threats to public health (Antimicrobial Resistance, 2022). The majority of *Kpn* strains are encapsulated by polysaccharide capsular material (CPS), a critical virulence determinant. Based on CPS variation, *Kpn* can be classified into at least 78 distinct serotypes (Zhao R. et al., 2024). Genomic and epidemiological analyses of 1,649 *Kpn* isolates from 244 hospitals across 32 European countries revealed that carbapenemase production represents the primary mechanism driving carbapenem resistance across diverse phylogenetic lineages (David et al., 2019). Nonetheless, the vast majority of carbapenemase-producing isolates belong to only four major clonal lineages-sequence types (STs) 11, 15, 101, and 258/512-and their derivatives (David et al., 2019).

Bacteriophages (phages), naturally occurring viruses that specifically infect bacterial hosts, are categorized into lysogenic and lytic types based on their mechanisms of infection. Phage therapy provides a promising complement to antibiotics against multidrug-resistant (MDR) bacterial infections (Torres-Barcelo and Hochberg, 2016; Chegini et al., 2021; Fang et al., 2023; Zhao M. et al., 2024; Huang et al., 2025). In recent years, significant progress has been made in phage therapy for treating and preventing pathogenic bacterial infections, attracting increasingly widespread attention (Duan et al., 2022; Hatfull et al., 2022; Uyttebroek et al., 2022; Strathdee et al., 2023; Fang et al., 2024; Berryhill et al., 2025). Compared with antibiotic treatments, phage therapy remains efficacy against drug-resistant pathogens. Moreover, phage therapy can specifically target pathogenic bacteria while sparing the resident microbiota, thereby preserving the integrity of the host’s microbial community.

*Kpn* is a gram-negative pathogen that contain lipopolysaccharides (LPS), peptidoglycans, periplasmic, cytoplasmic proteins, capsular polysaccharide, and nucleic acids (Xu et al., 2024; Li et al., 2025). LPS was regarded as an important factor that induces inflammation among Gram-negative bacteria by promoting Toll like receptor 4 (TLR4) signaling pathway activation (Ryu et al., 2017). Attenuating TLR4 induced inflammation could alleviate *Kpn*-induced pneumonia (Wei et al., 2025). As a new approach to combat *Kpn*, phage therapy has exhibited positive clinical effects in some animal experiments and small-scale clinical studies (Federici et al., 2022; Li et al., 2023; Chen et al., 2024). However, the interactions among phage, *Kpn*, and *Kpn*−infected host cells warrant further investigation. In this study, we isolated a *Kpn*−specific bacteriophage and examined its capacity to mitigate *Kpn*−induced inflammation and cell death. We also assessed the phage’s therapeutic performance in a mouse model of *Kpn* infection.

## Materials and methods

### Cells

HEK293T, A549, THP-1 and Hela cells were obtained from the American Type Culture Collection. HEK293T, A549 cells and Hela cells were grown in Dulbecco’s modified Eagle medium (DMEM) supplemented with 10% fetal bovine serum (FBS) and 1% antibiotics (penicillin and streptomycin) at 37 °C in 5% CO2. THP-1 cells were grown in RPMI-1640 supplemented with 10% FBS and 1% antibiotics (penicillin and streptomycin) at 37 °C in 5% CO2.

### Phage isolation and purification

A novel lytic phage, named vB_KpnP-6K2 (6K2), was screened from hospital sewage samples using a ST11-KL64 type *Kpn* as the host bacterium. In brief, sewage samples were centrifuged at 4,000 × g for 10 min. The resulting supernatant was filtered through a 0.22 µm membrane (Millex-GP Filter Unit, Millipore, USA). Subsequently, 5 mL of the filtrate was combined with 5 mL of double-strength LB broth supplemented with host bacteria and incubated overnight at 37 °C. After incubation, the mixture was centrifuged at 4,000 × g for 10 min, and the supernatant was again filtered through a 0.22 µm filter. The filtrate was serially diluted in LB medium, and plaques were observed using the double-layer agar method. The purification procedure was repeated at least three times until homogeneous phage plaques were observed. Finally, SM buffer containing purified phage was centrifuged for 10 min at 4,000× g and filtered through a 0.22 µm syringe-driven filter. The supernatant was added with 10% glycerol and stored at -80 °C (Garcia-Cruz et al., 2023; Abdel-Razek et al., 2025; Mirza et al., 2025).

### Multiplicity of infection assay

To determine the optimal multiplicity of infection (MOI) for maximizing phage progeny yield, the host strain *Kpn* was grown to logarithmic phase and adjusted to a turbidity equivalent to 0.5 McFarland standard (approximately 10^8^ CFU/mL). A volume of 10 mL of the host bacterial suspension at 10^7^ CFU/mL was mixed with 10 μL of serially diluted phage suspensions (approximately 10^7^, 10^6^, 10^5^, 10^4^, 10^3^, 10^2^, 10^1^, and 10^0^ PFU/mL), as previously described (Zhou et al., 2018). After 6 hours of incubation, the number of progeny phages was quantified using the double-layer agar method.

### pH and thermal stability

We evaluated the stability of phage under varying pH conditions and temperatures as previously described (Fang and Zong, 2022). Phage suspensions (100 μL) at a concentration of 10^9^ PFU/mL were mixed with 900 μL of SM buffer adjusted to different pH values ranging from 2 to 13. After incubation at 37 °C for 1 hour, the phage titer was quantified using the double-layer agar method. Thermal stability was assessed by incubating phage suspensions at -20, 4, 37, 50, 60, and 70 °C for 1 hour in a water bath, followed by titer determination as described above. All experiments were conducted in triplicate.

### Transmission electron microscopy

We observed the morphology and the size of phage 6K2 using transmission electron microscopy (TEM) imaging. Lysates of phage 6K2 were centrifuged at 16,000× g for 10 min and supernatants were filtered through 0.22 µm to remove bacteria and cell debris. This procedure yielded a final phage titer of 1 × 10^9^ PFU/mL. We dropped the phage suspension to copper grids for 10 min and then added a drop of phosphotungstic acid (Solarbio, Cat: G1872) for negative staining. We used a Hitachi Transmission Electron Microscope (Hitachi High-Tech; Tokyo, Japan) at an accelerating voltage of 80 kV for TEM.

### Phage adsorption and one-step growth

We assessed the phage adsorption capability by combining 10 mL of the *Kpn* (10^8^ CFU/mL) with 100 μL of phage suspension (10^8^ PFU/mL) at a MOI of 0.01. Samples were collected at 0-, 3-, 6-, 9-, 12-, and 15-min post-infection and immediately filtered through 0.22 µm membranes to determine the titers of unabsorbed phages. Host bacteria at the mid-log phase were infected with phages at an MOI of 0.01 and adsorbed for 10 min at 37 °C. After removing unabsorbed phages by centrifugation, the infected cells were resuspended in fresh medium to a 10-fold dilution. The diluted culture was incubated at 37 °C with shaking, and samples were collected at indicated time points. Each sample was immediately serially diluted and assayed for PFU using the double-agar overlay method.

### Phage genome sequencing, assembly, and annotation

The DNA of phage 6K2 was extracted using a Viral Genome Extraction Kit (Tiangen, China) and sent to Chengdu Phagetimes Biotech Co., Ltd., and the genome was sequenced using an Illumina Novaseq 6000. Raw sequencing reads were processed for quality control and adapter trimming using fastp (Chen et al., 2018). This step removed low-quality reads and reads with a high proportion of ambiguous bases (N), yielding high-quality clean reads. *De novo* assembly of the clean reads was performed with metaSPAdes (Nurk et al., 2017). Based on the assembled contig sequence, specific primers spanning the terminal region were designed to verify the genome is circular or not (Boeckman et al., 2024). The primer sequences used were 6K2-end-F: 5’-CGTATGTCCGGTTGATGACTAC-3’ and 6K2-end-R: 5’- CTATGCACTGACCTGAGGATTAC-3’. Following PCR amplification, the product was purified and its sequence was determined by Sanger sequencing.

The assembled genome was annotated for protein-coding genes and tRNAs using the RAST server (https://rast.nmpdr.org/), and the annotation was further verified by Blastp. Potential virulence factors and antimicrobial resistance genes were identified by screening the genome sequences against the Virulence Factor Database (VFDB) and the ResFinder database, respectively. Finally, the phage genome was visualized using an online Circos tool (https://www.chiplot.online/circos.html).

### Assessment of apoptosis by flow cytometry

A549 cells were seeded in 12-well plates at a density of approximately 1×10^5^ cells per well. After 12 hours, the culture medium was replaced with DMEM supplemented with 10% FBS and without antibiotics. The cells were then infected with 5 μL of *Kpn* containing approximately 1×10^6^ CFU, while the control group received an equivalent volume of sterile LB medium. Following co-culture for 12 and 24 hours, cells were harvested and stained by Cell Cycle and Apoptosis Analysis Kit (Yeasen, 40301ES50) to analyze the apoptosis by flow cytometry (Crowley et al., 2016).

### Detection of inflammatory cytokine and chemokine expression by qPCR

Total RNA was extracted using TRIzol reagent (Rio et al., 2010) and reverse-transcribed to cDNA for qPCR analysis to measure mRNA levels of the indicated genes. The mRNA levels of the tested genes were normalized to 18S rRNA levels. Gene-specific primer sequences were as follows:

*18S*: CCGGTACAGTGAAACTGCGAATG (forward) and
GTTATCCAAGTAGGAGAGGAGCGAG (reverse),
*IL1B*: CCACAGACCTTCCAGGAGAATG (forward) and
GTGCAGTTCAGTGATCGTACAGG (reverse),
*IL6*: AGACAGCCACTCACCTCTTCAG (forward) and
TTCTGCCAGTGCCTCTTTGCTG (reverse),
*TNFA*: CTCTTCTGCCTGCTGCACTTTG (forward) and
ATGGGCTACAGGCTTGTCACTC (reverse),
*CXCL1*: AGCTTGCCTCAATCCTGCATCC (forward) and
TCCTTCAGGAACAGCCACCAGT (reverse),
*CXCL2*: GGCAGAAAGCTTGTCTCAACCC (forward) and
CTCCTTCAGGAACAGCCACCAA (reverse),
*CXCL3*: TTCACCTCAAGAACATCCAAAGTG (forward) and
TTCTTCCCATTCTTGAGTGTGGC (reverse),
*CXCL5*: CAGACCACGCAAGGAGTTCATC (forward) and
TTCCTTCCCGTTCTTCAGGGAG (reverse),
*CXCL8*: GAGAGTGATTGAGAGTGGACCAC (forward) and
CACAACCCTCTGCACCCAGTTT (reverse),
*CXCL10*: GGTGAGAAGAGATGTCTGAATCC (forward) and
GTCCATCCTTGGAAGCACTGCA (reverse),
*CXCL12*: CTCAACACTCCAAACTGTGCCC (forward) and
CTCCAGGTACTCCTGAATCCAC (reverse),
*IFNB*: GACAGGATGAACTTTGACATCCC (forward) and
CTCAACAATAGTCTCATTCCAGCC (reverse),
*ISG54*: GGAGCAGATTCTGAGGCTTTGC (forward) and
GGATGAGGCTTCCAGACTCCAA (reverse).

### Mice experiments

Six-week-old male C57BL/6J mice were divided into seven groups (n = 10 per group) and intraperitoneally (IP) injected with 200 μL of *Kpn* at doses of 5 × 10^5^, 1 × 10^6^, 5 × 10^6^, 1 × 10^7^, 1 × 10^7^, 5 × 10^7^, or 1 × 10^8^ CFU. Control mice received an equal volume of PBS. Survival and body weight changes were monitored over time.

In an independent experiment, six-week-old male C57BL/6J mice were allocated into six groups and intraperitoneally challenged with 200 μL of *Kpn* at 1 × 10^8^ CFU (n ≥ 5 per group) or with PBS (n =3 per group). 1h later, 6K2 was administered at MOI of 0, 1 and 10. Mice in the MOI = 0 group received an equivalent volume of PBS as control. Survival was recorded every 3 h for all mice. In the *Kpn*−infected group, blood samples were collected at 1, 6, and 24h post phage administration to quantify bacterial load (CFU/mL) by plate counting and phage titers (PFU/mL) using the double-layer agar method. For mice not infected with *Kpn*, blood samples were collected solely for monitoring phage titer changes by the double−layer agar assay. All animal experiments were carried out in Jinan Microecological Biomedicine Shandong Laboratory (Jinan, China) according to procedures approved by the institutional ethics committee (Animal testing approval number:2026001).

### Statistical analysis

Statistical significance was calculated using an unpaired Student’s t test (two-tailed). Results are shown as arithmetic means ± SD of at least 3 or more independent measurements. GraphPad Prism 9 was used for statistical analysis.

## Results

### Morphology of phage 6K2

**Figure 1:**
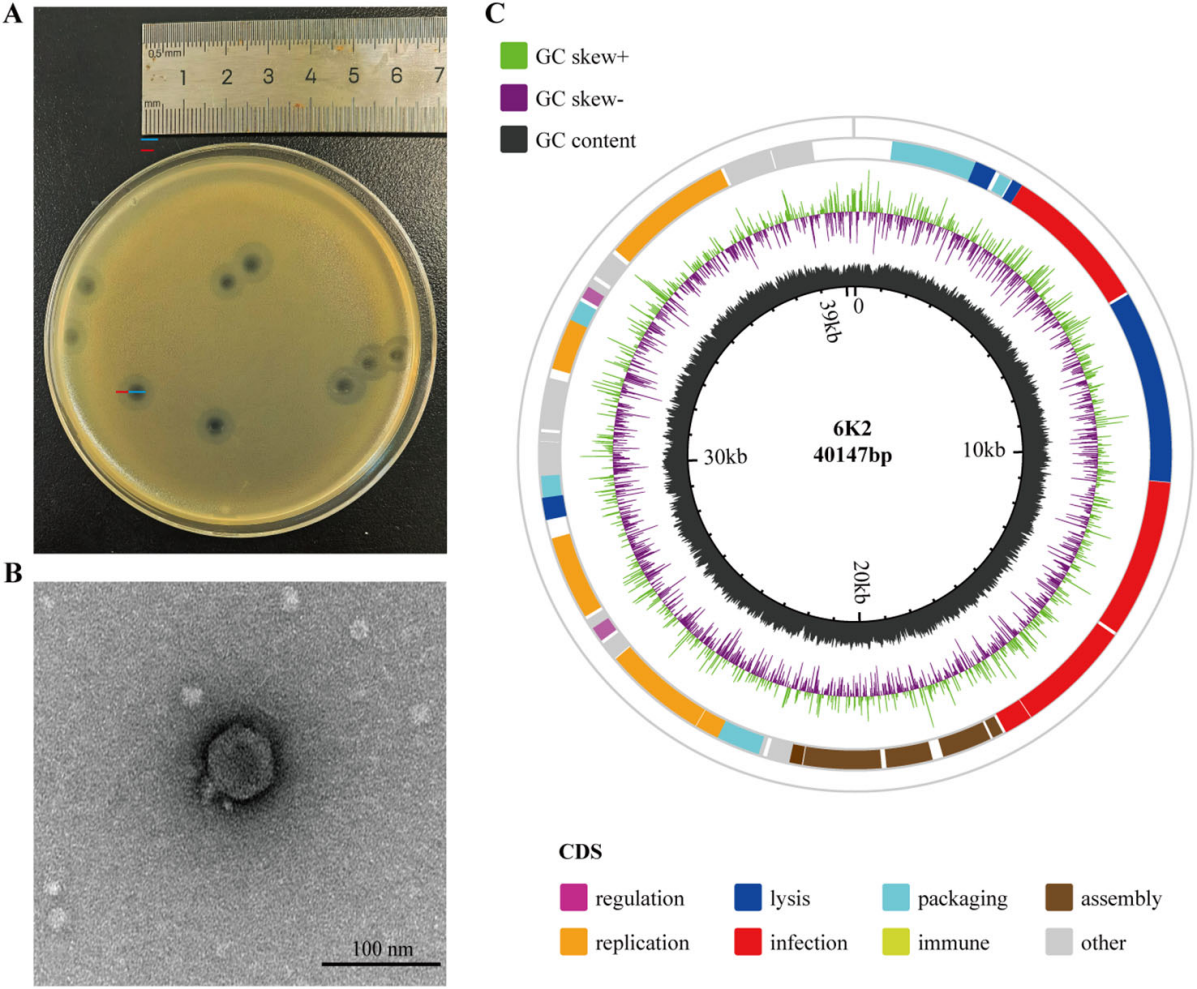
Morphology of phage 6K2. **(A)** Plaques formed by phage 6K2 on *Kpn* host using the double agar overlay method. **(B)** Transmission electron microscopy (TEM) image of phage 6K2. The head diameter was approximately 60 nm, with a tail measuring 15 nm in length and 15 nm in width. Scale bar: 100 nm. **(C)** Genomic maps of phage 6K2. Genome was annotated using the RAST server. ORF positions are represented by colored bars on concentric rings, with the specific ring (inner or outer) indicating the direction of transcription. Functional categories are distinguished by color, as detailed in the legend. The circular genome map was generated using an online Circos tool (https://www.chiplot.online/circos.html).

The bacteriophage 6K2 was isolated from hospital sewage using a ST11-KL64 type *Kpn* as the host bacterium. Purified phage 6K2 formed clear plaques (the diameter about 4 mm) with translucent halos (the diameter about 3 mm) on double-layer agar plates (**Figure 1A**). Transmission electron microscopy (TEM) revealed that bacteriophage 6K2 had a polyhedral symmetric head (the head diameter was approximately 60 nm) and a short non-contractile tail (the tail length was 15 nm and the width was 15 nm), which is a typical morphology of the family *Podoviridae* (**Figure 1B**).

### Genome features and annotation

*De novo* assembly of the 6K2 genome produced a single contig of 40,328 bp, which featured terminal repeats of 181 bp at both ends. To determine whether this structure represented a linear genome with redundant termini or a circular conformation, we performed PCR with primers spanning the repeat junction. Sanger sequencing of the amplicon confirmed a circular genome structure. The complete, circularized double-stranded DNA genome comprises 40,147 bp with a GC content of 53.05% (**Figure 1C**; see Additional file 1 for sequence verification details). Phage 6K2 has 48 predicted open reading frames (ORFs), with 32 ORFs (68.1%) annotated with predicted functions (Additional file 2). The annotated genes of 6K2 were categorized into different functional groups, including phage infection, DNA replication and regulation, phage capsid assembly, DNA packaging and host lysis (**Figure 1C**). In addition, no homologs of known virulence factors or antibiotic resistance genes were identified in the 6K2 genome, suggesting its potential suitability for clinical applications.

**Figure 2:**
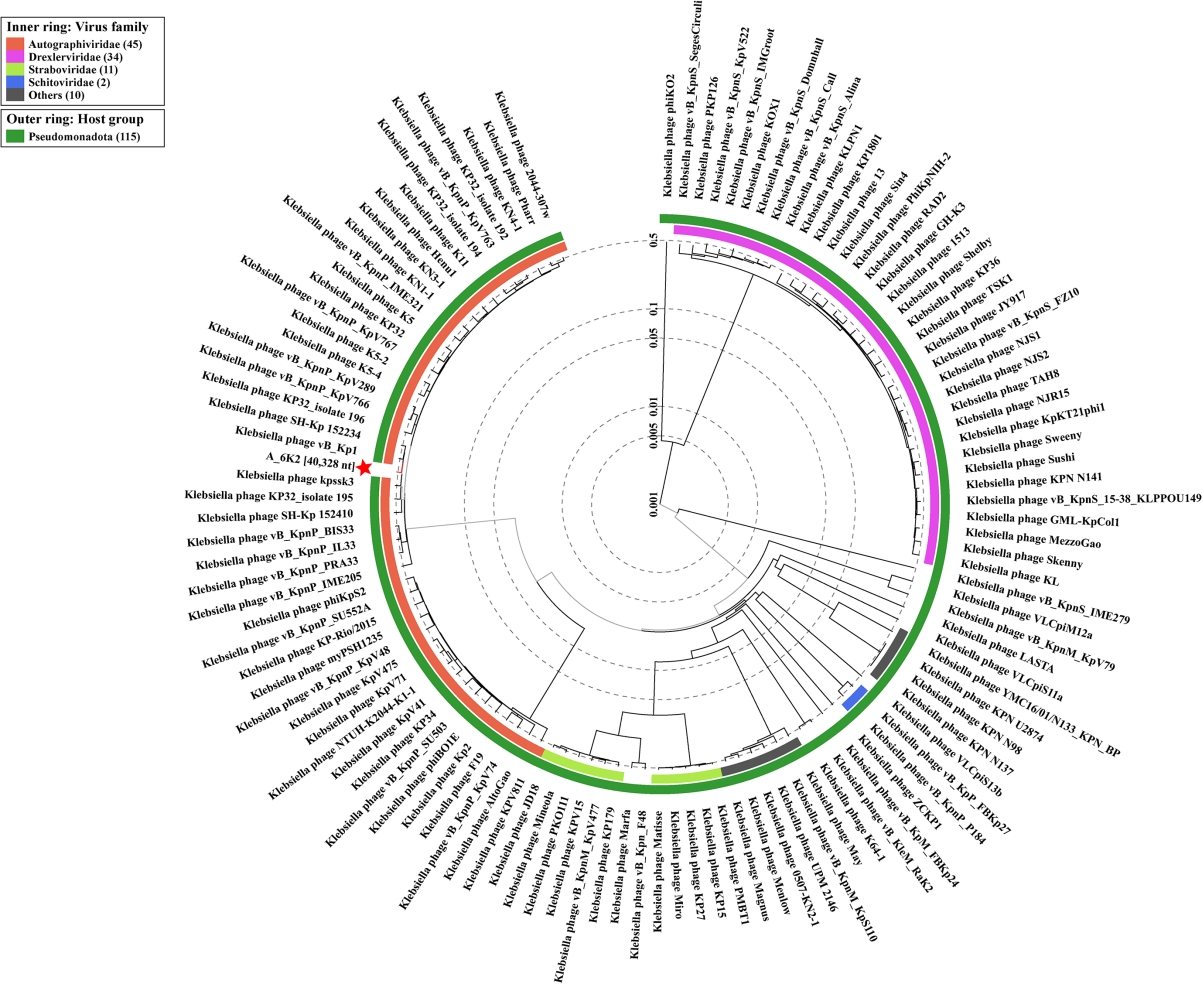
Phylogenetic tree of 6K2 and relative bacteriophages based on the complete genome sequences. The tree was constructed using the ViPTree. The red star indicates the phage 6K2.

The phylogenetic relatedness of 6K2 with similar phages was determined based on genome-wide sequence similarities calculated by tBLASTx with the use ViPTree (https://www.genome.jp/viptree/). 6K2 was clustered with *Klebsiella* phage vB *Kpn*16-P1, vB *Kpn*16-P3, vB Kp IME531 and Kp 11 (**Figure 2**). Based on these results and the viral classification provided by the International Committee on Taxonomy of Viruses (ICTV), it was concluded that 6K2 belongs to the family of *Autographiviridae*, within the genus of *Przondovirus*.

### Characterization of phage 6K2

**Figure 3:**
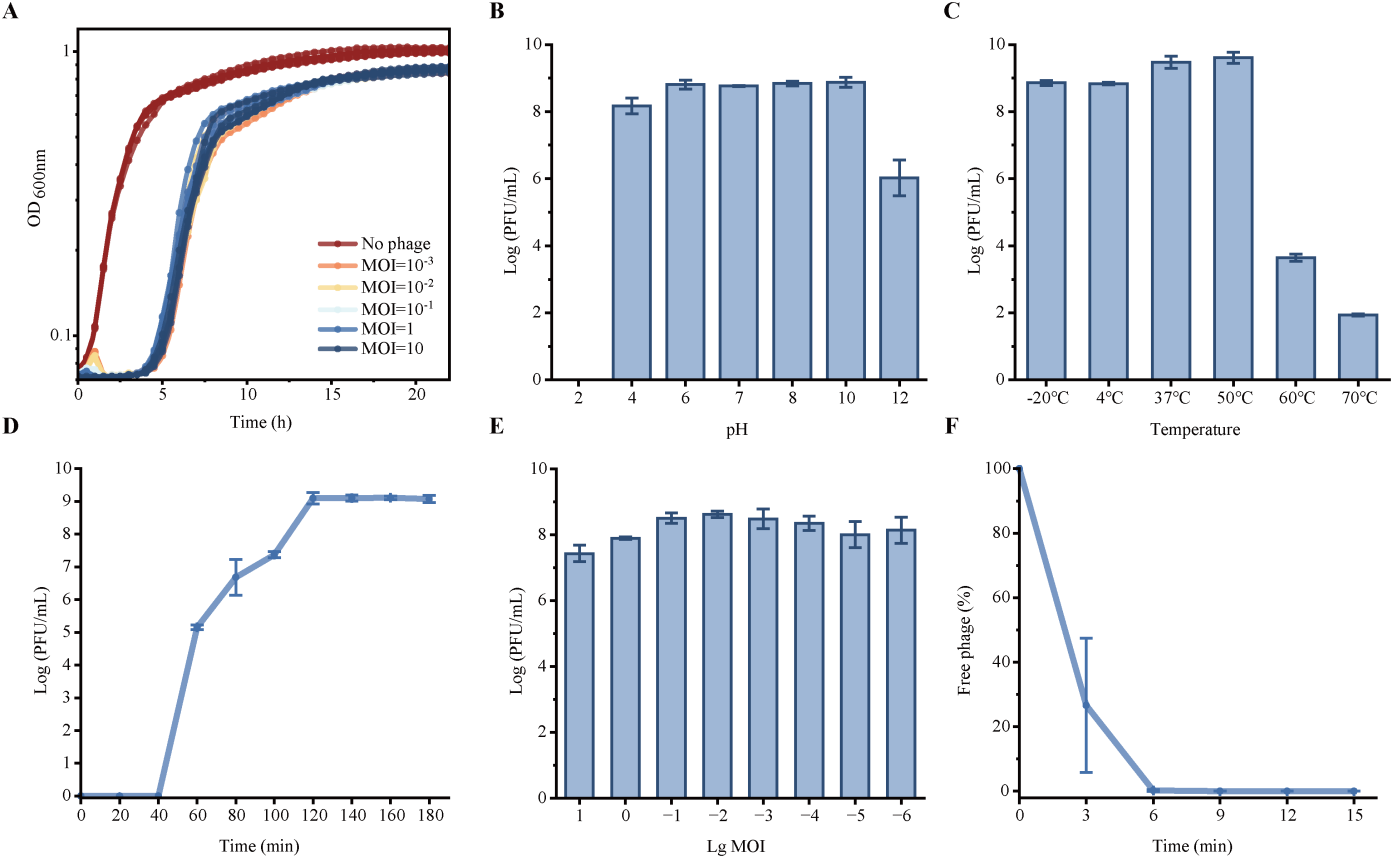
Characterization of phage 6K2. **(A)** The bacteriolytic activity of 6K2 *in vitro*. **(B)** pH stability detection of 6K2. **(C)** Temperature stability of 6K2. **(D)** One-step growth curve of 6K2. **(E)** The optimal multiplicity of infection. **(F)** Absorption rate of 6K2.

To determine the efficiency of phage 6K2 in killing the *Kpn*, we examined its inhibitory effects at varying doses. Complete inhibition of *Kpn* by 6K2 was observed at MOIs ranging from 0.001 to 10 (**Figure 3A**). During the first 2 hours post-infection, the group of 6K2 (MOI≥0.1) effectively suppressed *Kpn* growth. After this initial period, however, bacterial loads in groups with an MOI <0.1 also became suppressed, resulting in no significant difference compared to the higher MOI groups. The results of pH and thermal stability showed that 6K2 has a wide pH tolerance, which is ranging from 4 to 12 (no significant differences among the four groups tested at pH 6, pH 7, pH 8, and pH 10) (**Figure 3B**). Although with minimal loss in titer, 6K2 remained active between 4 °C and 50 °C. However, the phage titer of 6K2 decreased by 5 log and 7 log after incubation of 1 hour at 60 °C and 70 °C, respectively (**Figure 3C**). No viable phage particles were detected following treatment at 80 °C (data not show). This suggests that phage 6K2 is less tolerant to the temperatures above 60 °C. The one-step growth curve analysis demonstrated the latent period of 6K2 lasted for about 40 min and followed by a burst period lasted for about 80 min (**Figure 3D**). And the burst size was calculated as 13.6 PFU per host cell. The optimal multiplicity of infection (MOI) was 0.01 of 6K2 vs *Kpn* (**Figure 3E**). Phage adsorption measurements showed that about 80% phages absorbed to host cells at 3 min and almost all phages absorbed to host cells after 6 min (**Figure 3F**).

### Monocyte is responsible for the inflammation after *Kpn* infection

**Figure 4:**
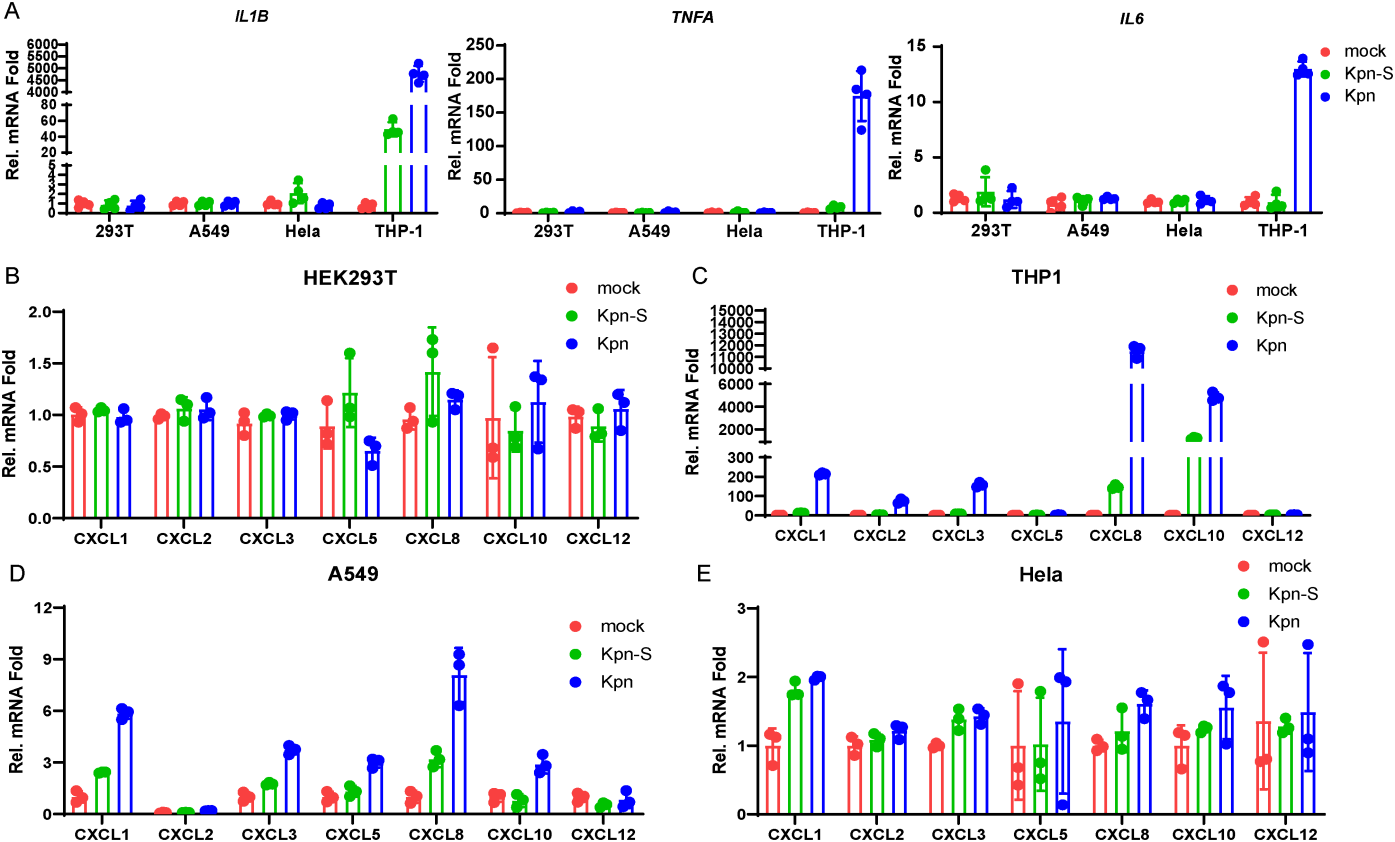
Inflammatory responses in various cell types induced by *Kpn*. **(A)** HEK293T, A549, Hela, THP-1 cells were treated with *Kpn* and its culture supernatant for 12h. the cells were collected for qPCR assay to detect the *IL1B*, *TNFA*, and *IL6* expression. **(B–E)** HEK293T, A549, Hela, THP-1 cells were treated with *Kpn* and its culture supernatant for 12h. the cells were collected for qPCR assay to detect the *CXCL1, CXCL2, CXCL3, CXCL5, CXCL8, CXCL10*, and *CXCL12* expression. mock: cells were treated with an equal volume of LB medium.

Infections caused by *Kpn* encompass a broad spectrum of clinical manifestations, including renal impairment in kidney transplant recipients, pulmonary infections leading to pneumonia, bloodstream and intra-abdominal infections resulting in sepsis (Yang Y. et al., 2021; Zhang et al., 2021; Zheng et al., 2022; Tang et al., 2024; Yang YY. et al., 2024). Therefore, we assessed the ability of *Kpn* and its culture supernatant to induce inflammatory responses in various cell types. Our results demonstrated that *Kpn* and its culture supernatant treatment only promote *IL1B*, *TNFA* and *IL6* expression in THP-1 (**Figure 4A**). During the initial phase of an infection, resident cells release chemokines that recruit diverse immune cells to the site of infection, where they exert their effector functions (Borish and Steinke, 2003; van der Vorst et al., 2015). Furthermore, we also assessed the chemokines expression induced by *Kpn* and its culture supernatant. Treatment with *Kpn* or its supernatant upregulates chemokine expression in THP-1 and A549 cells, but not in HEK293T or HeLa cells (**Figures 4B-E**). Notably, *CXCL5* and *CXCL10* induction was absent in THP-1 cells (**Figure 4C**), while *CXCL2* and *CXCL12* were not induced in A549 cells (**Figure 4D**).

### 6K2 reduces cytokines and chemokine expression during *Kpn* infection

**Figure 5:**
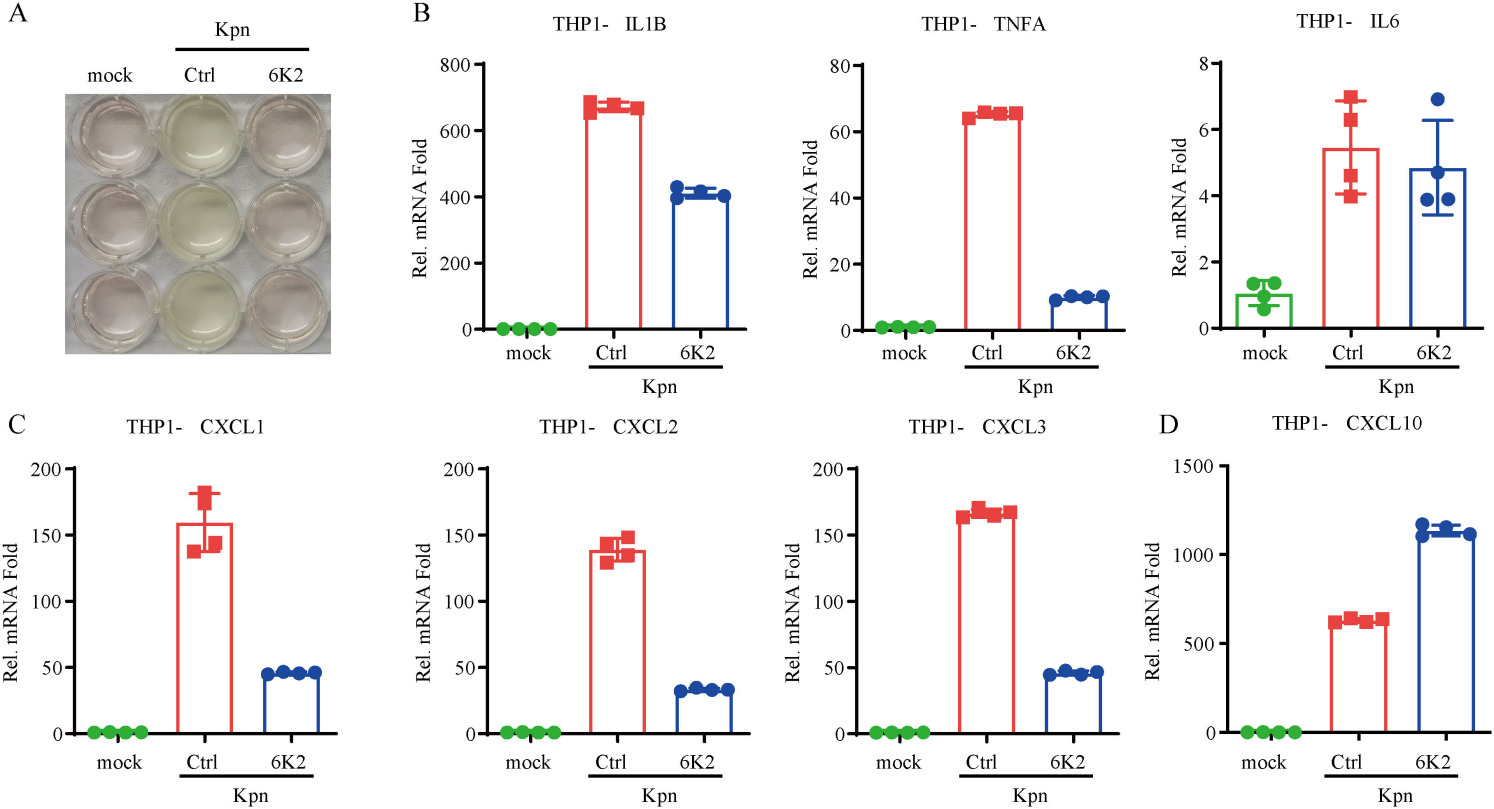
6K2 Reduces cytokines and chemokine expression during *Kpn* infection. **(A)***Kpn* and THP-1 cells were co-cultured in RPMI 1640 supplemented with 10% FBS at 37 °C for 6 h, and the culture supernatant was subsequently imaged. **(B–D)***Kpn* and THP-1 cells were co-cultured under the same conditions in the presence or absence of 6K2 for 12 h, after which cells were harvested for qPCR to analysis the cytokines **(B)** and chemokine **(C, D)** expression. mock: cells were treated with an equal volume of LB medium and SM buffer.

After 6 hours incubation of *Kpn* and THP-1 at 37 °C, cell culture medium (RPMI 1640 cell culture medium with 10% FBS) exhibited acidification. Treatment with 6K2 significantly alleviated the medium acidification induced by *Kpn* infection (**Figure 5A**). Acidification of the cell culture medium serves as an indicator of *Kpn* proliferation. In order to determine 6K2 on the inflammation and chemokine expression induced by *Kpn* infection. qPCR assay was performed, our results demonstrated that 6K2 markedly suppressed the *IL1B* and *TNFA* expression significantly but not *IL6* (**Figure 5B**). Moreover, 6K2 suppressed the expression of chemokines (*CXCL1*, *CXCL2*, and *CXCL3*) following *Kpn* infection (**Figure 5C**). In contrast, our data demonstrate that 6K2 potentiated the upregulation of *CXCL10* in response to *Kpn* infection (**Figure 5D**).

### 6K2 promotes antiviral innate immunity and inflammatory response activation

**Figure 6:**
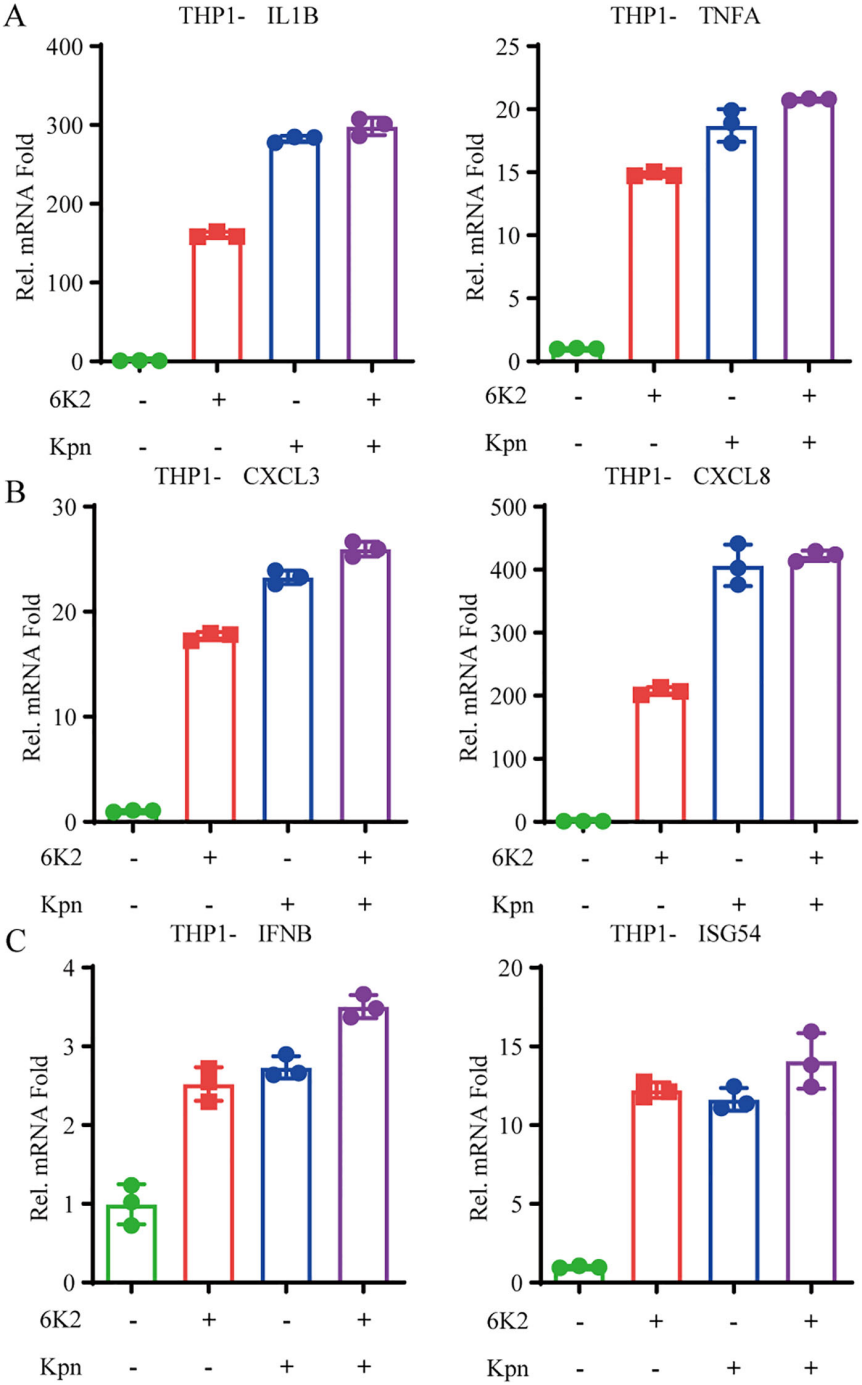
6K2 promotes antiviral innate immunity and inflammatory response activation. **(A-C)** THP-1 cells were treated with heat-killed *Kpn* and bacteriophage 6K2, while control groups received equivalent volumes of LB medium and PBS, respectively. After 12 hours, cells were harvested, and the expression of inflammatory cytokines (*IL1B, TNFA*) **(A)**, chemokines (*CXCL3, CXCL8*) **(B)**, and interferon pathway antiviral genes (*IFNB, ISG54*) **(C)** was analyzed by qPCR.

To further investigate whether 6K2 inhibits inflammation by clearing Klebsiella pneumoniae infection or exerts direct anti-inflammatory effects, we treated THP-1 cells with heat-killed *Kpn* to induce inflammation. We found that 6K2 did not suppress the expression of inflammatory cytokines and chemokines induced by heat-killed *Kpn*. Moreover, 6K2 itself could also induce the expression of inflammatory cytokines and chemokines (**Figures 6A, B**). To determine whether phage 6K2 acts as a viral agent that triggers interferon-mediated antiviral immune responses, we performed qPCR analysis and observed that 6K2 induced the expression of *IFNB* and interferon-stimulated gene *ISG54* (**Figure 6C**).

### 6K2 inhibits cell death induced by *Kpn* infection

**Figure 7:**
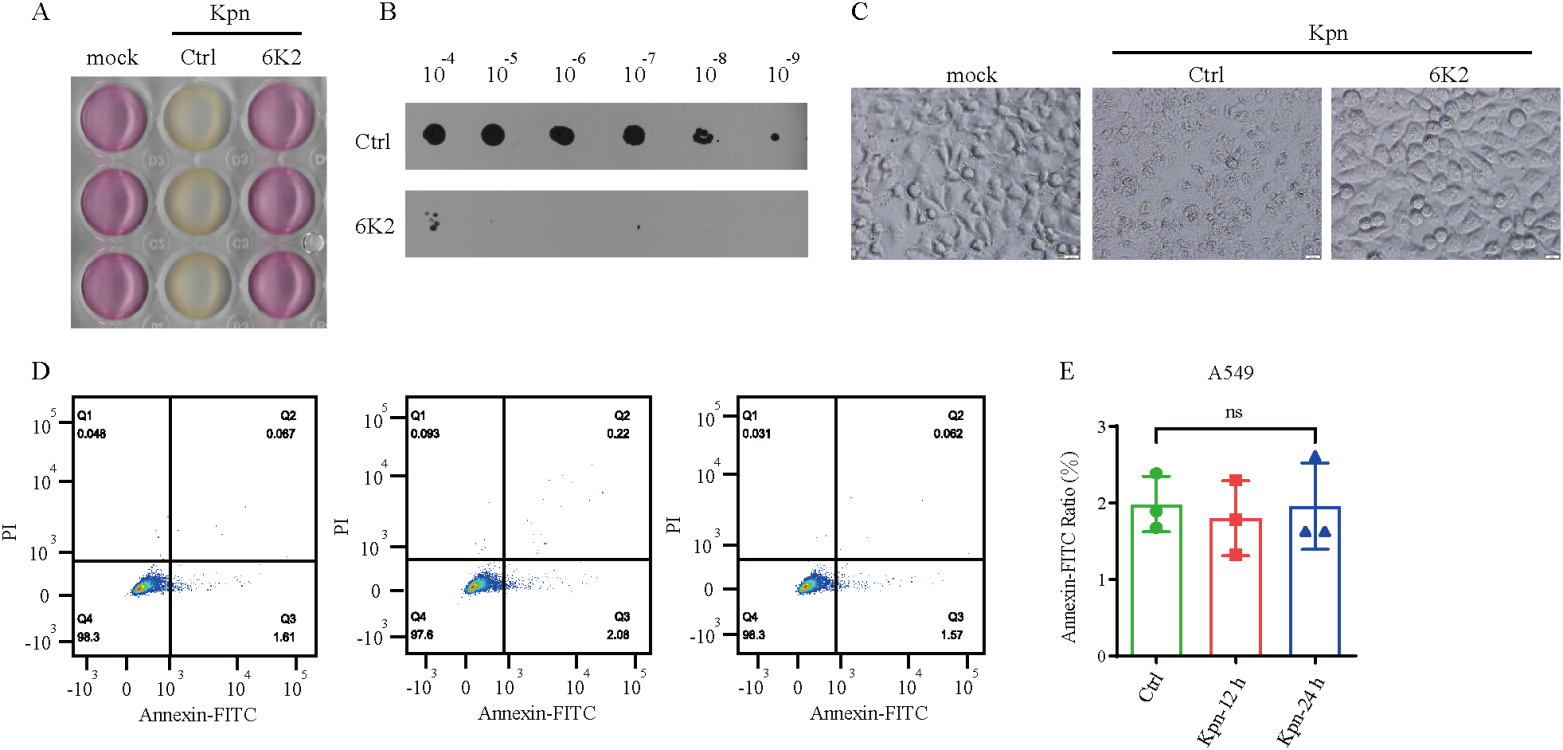
6K2 inhibits cell death induced by *Kpn* infection. **(A–D)***Kpn* and A549 cells were co-cultured under the same conditions in the presence or absence of 6K2 for 6 h, after which the following performants were conducted. **(A)** imaging was conducted to record the changes in the co-culture system. **(B)** the CFU of *Kpn* was analyzed. **(C)** morphological changes of A549 were conducted by microscopic examination. **(D)** A549 apoptosis were analyzed by flow cytometry. **(E)** Quantification of A549 apoptosis rates.

Previous studies have demonstrated that *Kpn* infection could induce cell death by promoting apoptosis, pyroptosis, and autophagy (Wang et al., 2018; Jiang et al., 2022; Wang et al., 2023; Wei et al., 2023). Therefore, we further investigated the impact of *Kpn* infection on cell death. Initially, co-culture of *Kpn* with A549 cells was found to promote acidification of the culture medium, while 6K2 inhibited this *Kpn*-induced acidification (**Figure 7A**). Further examination of the *Kpn* in the co-culture system revealed that 6K2 significantly reduced *Kpn* proliferation (**Figure 7B**). Microscopic observation of A549 cell morphology showed that *Kpn* induced cell rounding, fragmentation, and death, whereas 6K2 markedly alleviated these morphological changes and reduced cell death (**Figure 7C**). To further elucidate the mode of *Kpn*-induced A549 cell death in the co-culture system, flow cytometry analysis was performed. The results indicated that *Kpn*-induced A549 cell death did not occur via apoptosis (**Figures 7D, E**).

### 6K2 protects against lethal *Kpn* infection *in vivo*

**Figure 8:**
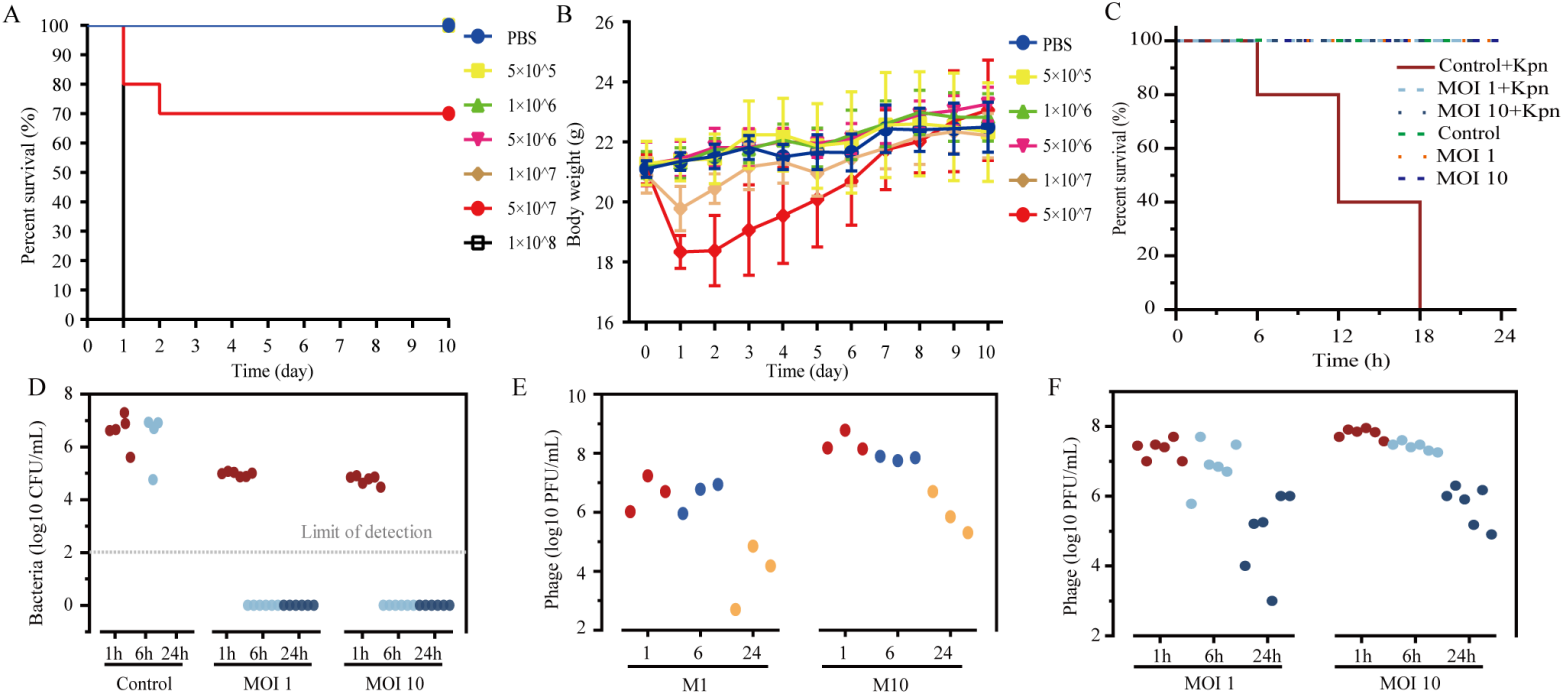
Prompt phage treatment rescues mice with systemic *Kpn* infection. **(A)** Survival of mice after a single IP injection with the indicated inoculum (CFU) of *Kpn*. n = 10. **(B)** The weight of surviving mice was determined every day. **(C)** Survival of mice infected IP with *Kpn* (1×10^8^ CFU) followed by IP treatment with phage (MOI = 1, 10) 1h later. Equivalent volumes of PBS were injected in lieu of phage as control. For each group, For the *Kpn*-infected groups, n ≥ 5; for the groups injected with bacteriophages only, n = 3. **(D)** Blood was collected via the tail vein at 1, 6, and 24h post-phage treatment to determine the CFU of *Kpn* in the blood. **(E, F)** Blood samples were collected from the tail vein at 1, 6, and 24 h after phage administration to quantify phage 6K2 PFU in the bloodstream of phage-only control mice **(E)** and *Kpn*-infected mice **(F)**.

To determine whether 6K2 could protect against lethal *Kpn* infection *in vivo*, we first established a murine model of bloodstream infection using *Kpn*. Our results showed that mice administered *Kpn* at doses exceeding 1×10^8^ CFU exhibited 100% mortality within 24 hours, while a dose of 5×10^7^ CFU led to approximately 70% mortality within two days, accompanied by a transient decrease in body weight, followed by gradual recovery (**Figures 8A, B**). Based on these findings, mice were infected with 1×10^8^ CFU of *Kpn* and treated with 6K2. During the mouse challenge and treatment experiment, the clinical symptoms of the mice were observed every 3h. The results demonstrated that all 6K2-treated mice survived, whereas all control mice died within 18h (**Figure 8C**). Blood samples collected at 1, 6, and 24h post 6K2 administration revealed that the bacterial load of *Kpn* in the blood of 6K2-treated mice fell below the detection limit as early as 6 hours after treatment (**Figure 8D**). Furthermore, quantification of 6K2 phage in the blood indicated high phage titers within 6 hours of treatment, which subsequently decreased by 24 hours (**Figures 8E, F**).

## Discussion

A dearth of treatment options for infections caused by multidrug-resistant bacteria has led to renewed interest in the therapeutic potential of phage. Although numerous phage therapy cases have culminated in positive clinical outcomes. There are still some obstacles to be overcome. In addition to addressing issues such as the development of bacterial resistance to phages, it is of great significance for future clinical applications of phages to elucidate whether bacteria, phages, and bacterial lysates within the infectious microenvironment can trigger more intense immune responses and lead to the death of infected tissue cells. Recent years have witnessed a growing body of research highlighting the potential of bacteriophages in clearing bacterial infections and mitigating inflammatory responses, as demonstrated by *in vitro* and *in vivo* studies on the interactions among phages, bacteria, and host cells (Shi et al., 2021; Chen et al., 2025; Weissfuss et al., 2025). This research has moved beyond the binary interplay between phages and bacteria, increasingly reflecting the actual scenario of phage therapy during infection. Thus, further elucidation of the molecular mechanisms underlying the tripartite interaction among bacteriophages, bacteria, and host cells will provide critical insights to guide future clinical applications of phage-based therapies.

Bacteriophages are viruses capable of infecting and lysing bacteria. It is well known that animal viruses, such as SARS-CoV-2 and influenza viruses, trigger the host’s antiviral innate immune response upon infecting host cells, primarily through viral nucleic acids such as DNA or RNA (Fu et al., 2021; Wu et al., 2021; Cao et al., 2023; Yang J. et al., 2024; Zhang et al., 2024). However, bacteriophages also contain nucleic acid components. While they are used to treat bacterial infections, it remains worth exploring whether they can trigger host antiviral innate immune responses. In our study, we found that bacteriophage treatment upregulates the expression of *CXCL10* in monocytes (**Figure 5D**). *CXCL10* is an interferon-induced cytokine (Mowat et al., 2021; Gervais et al., 2024), indicating that bacteriophages may activate the host antiviral innate immune response during application. Our further investigation revealed that both phages and bacterial lysates could activate the interferon signaling pathway and induce the expression of inflammatory cytokines and chemokines (**Figure 6**). Previous work has shown that two *Kpn* phages can suppress the inflammatory response induced by *Kpn* infection in bovine mammary epithelial cells (bMECs) *in vitro* (Shi et al., 2021). That study, however, did not explore whether phages themselves might act as inflammatory stimuli. This is an important consideration for the future use of bacteriophages in therapeutic contexts.

In our *in vitro* experiments, where phages and bacteria were co-cultured, the growth of phage-resistant mutant bacterial strains gradually outcompeted that of the wild-type strains as the bacteria mutated, leading to a loss of the phage’s lytic activity. However, in our animal model, phage therapy demonstrated remarkably significant therapeutic efficacy (**Figures 8C-F**), substantially inhibiting mortality in mice infected with *Kpn* and rapidly reducing the bacterial load in the blood within a short period. We did not observe the emergence of phage-resistant mutant bacterial strains *in vivo*. These phenomena differ from our *in vitro* findings, which may be attributed to the fact that, in the mouse infection model, in addition to the interaction between phages and bacteria, the mouse immune system-including immune cells (such as NK cells and macrophages) and antibodies-also contributes to the clearance of *Kpn*. When phage therapy is applied, the bacterial load of *Kpn* is rapidly reduced to a level that can be efficiently cleared by the host immune system. The remaining or mutant bacterial strains are subsequently eliminated through the synergistic action of the immune system, thereby achieving an optimal therapeutic outcome.

## Data Availability

The datasets presented in this study can be found in online repositories. The names of the repository/repositories and accession number(s) can be found in the article/**Supplementary Material**.
