# Supplementary material for: A lytic bacteriophage vB_KpnP-6K2 inhibits ST11-KL64 Klebsiella pneumoniae induced cell death and inflammatory response
Source: Front Cell Infect Microbiol. 2026 Feb 20;16:1749949. doi: 10.3389/fcimb.2026.1749949 (PMC12963264; doi:10.3389/fcimb.2026.1749949)

As shown in the sequence below (displaying 700 bp from each end of the assembled contig, with the intervening sequence omitted), the terminal regions contained a repeated 181-bp segment (indicated by blue underline). Primers were designed to flank this region (red, bold text; arrows denote extension direction). If the genome were linear, no amplification would occur. Sanger sequencing of the PCR product confirmed a circular genome structure and revealed that the 181-bp repeat was an artifact generated during the de novo assembly process, not a genuine terminal redundancy. Therefore, we conclude that the complete, circular dsDNA genome of 6K2 is 40,147 bp in length.

>6K2 40328bp

GGGGACACAGAGACATCAACATATAGTCACCCAAGGTCACCCTCACCACAACATATAGTATCACCTAAGGGATTCCCAAGGTACCGACCCAAGGTTAAACCGAAGGTTTAGGGGTGGCCTATGGTTACTTTGGGTGAACTGGAGGGTACCGGGGGGATAACCAAAAGTGTAAACTGTGAGATGTGCACTCAGAACTTTGTGTAATATTCTTAAAGGCAACCTCAG**GTAATCCT**

6K2-end-R

**CAGGTCAGTGCATAG**ACCCGTAGGTAGACCCTGTGAATCACCTAAGGTTAACTTTAAGTATTGACTATTGAGGGATGGAGTGGTGTATGCTGGTAAGCATCACTACGGAATCCCTAGCGCGTCAGGAAGACCCTAATCGCTACAAGTGAGTAGAGAGCACACGAGAGTCTCCAGTCCACTGAGTTGCTGCTGAGTAACCAGTGAAGCCCCAAGGGCACCAGCAAGTACCAGCAGAAATCGCCAAGTAGTCCTATGGCGCAGTAAGGTTAACAATAAGCGCATAGGCCCTCCTTATGTTGGCTCTTAGTGTCTTATAGTTAGAGGGTGATATCATCATCACTACCCTCTCTCATAGAGGAGACTTAGAGTGCATAACTATATGAATGAAACTTTAAGTAGTCTTATAGTAAGTCTTTAGGGGTCTCTCCCTATAGTGCTACCTAATTCCAAGTGTCTGTTATACATAG∙∙∙∙∙∙∙∙∙∙∙∙∙∙∙∙∙∙∙∙∙∙∙∙∙∙∙∙∙∙∙∙∙∙∙∙∙∙∙∙∙∙∙∙∙∙∙∙∙∙∙∙∙∙∙∙∙∙∙∙∙∙∙∙∙∙∙∙∙∙∙∙∙∙∙∙∙∙∙∙∙∙∙∙∙∙∙∙∙∙∙∙∙∙∙∙∙∙∙∙∙∙∙∙∙∙∙∙∙∙∙∙∙∙∙∙∙∙∙∙∙∙∙∙∙∙∙∙∙∙∙∙∙∙∙∙∙∙∙∙∙∙∙∙∙∙∙∙∙∙∙∙∙∙∙∙∙∙∙∙∙∙∙∙∙∙∙∙∙∙∙∙∙∙∙∙∙∙∙∙∙∙∙∙∙∙∙∙∙∙∙∙∙∙∙∙∙∙∙∙∙∙∙∙∙∙∙∙∙∙∙∙∙∙∙∙∙∙∙∙∙∙∙∙∙∙TTCATCGTTGAGTCAACCACTTT**CGTATGTCCGGTTGATGACTAC**TTGAGACCCTCAGTCTAACCAGATAA

6K2-end-F

CTCGTGGTATTGTCTGGTTGTTGGTGACGTTGTGTCTGTCTCAACGGTTGCTAATGTCTCATAACGAAATCCGAATGTCAACACTTAAAGTTAAACTTTTAGTTAGACCTATAGTGATGGTGTTCTTATTGGTAATGGTCTCTTAGTAATACTTTAAGTGTCTCCTTATAGTGATACCTAATTGGTTGGTGTATTGACAATGACCACCAATAGCCCTTATAGTAATGGCTCACCGATACCATTTGTCCCGCTCTCAGTGTCTCAGGGACTGCTAAACGAGATACTTAATTGATTACCCTATGTGATCTACTGACAGTCACTGCTAAACGTTAGTAAAACGGTGAACCTAATGAGCTGTCTGCTCACCTAAGGTATATGGTCTCAGGTATTACCTCAGGTCGTTACCTCAGGAGGCCCTAAGGTGTGACTTAAAGAGGGCCAACAGATAGGGGACACAGAGACATCAACATATAGTCACCCAAGGTCACCCTCACCACAACATATAGTATCACCTAAGGGATTCCCAAGGTACCGACCCAAGGTTAAACCGAAGGTTTAGGGGTGGCCTATGGTTACTTTGGGTGAACTGGAGGGTACCGGGGGGATAACCAAAAGTGTAAACTGTGAGA

Sequencing result:


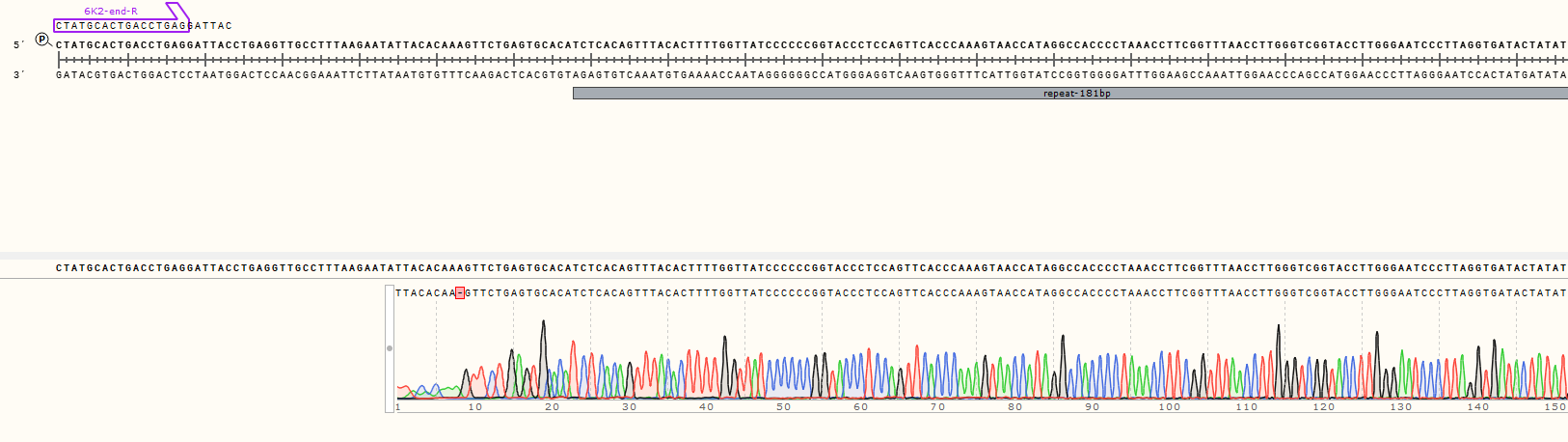


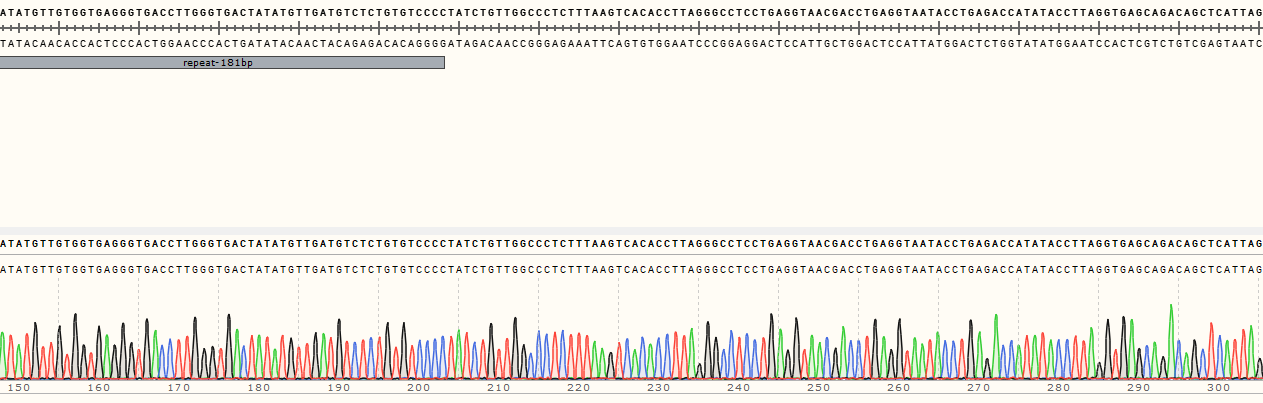


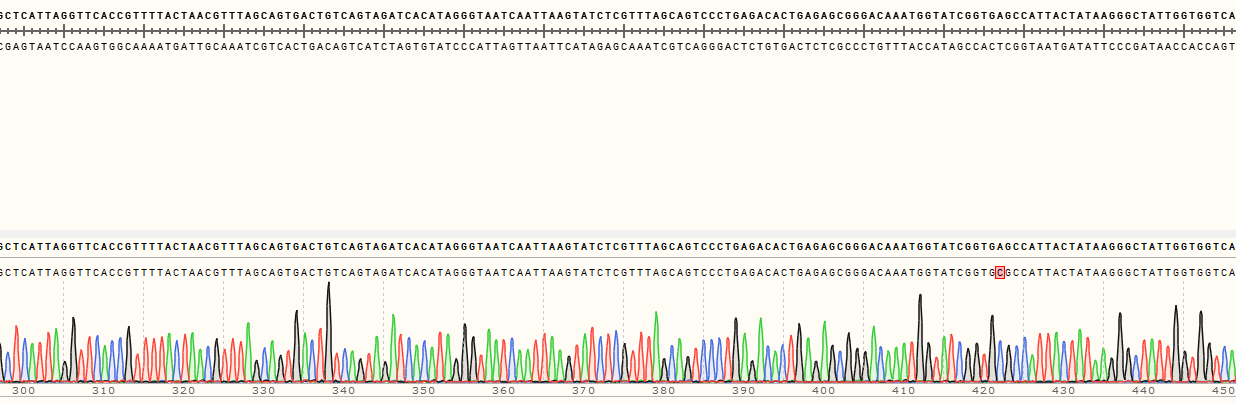


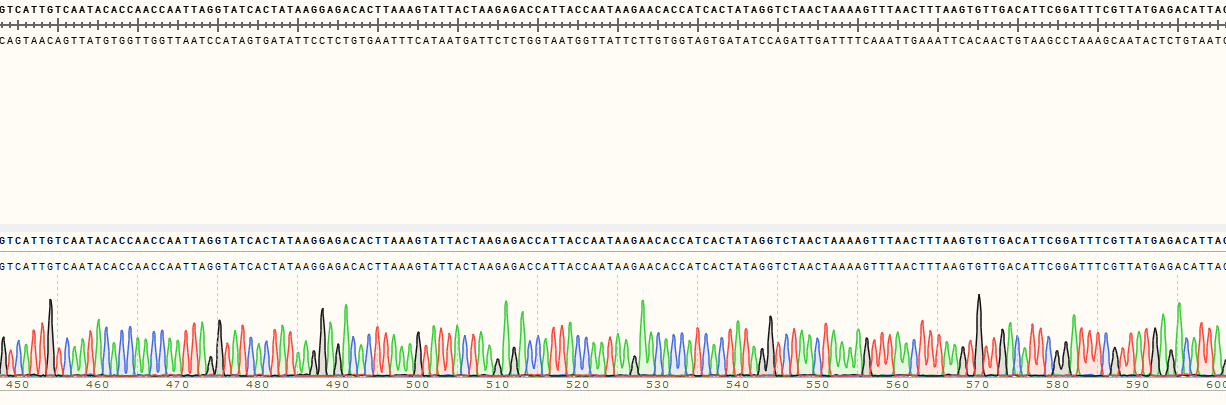


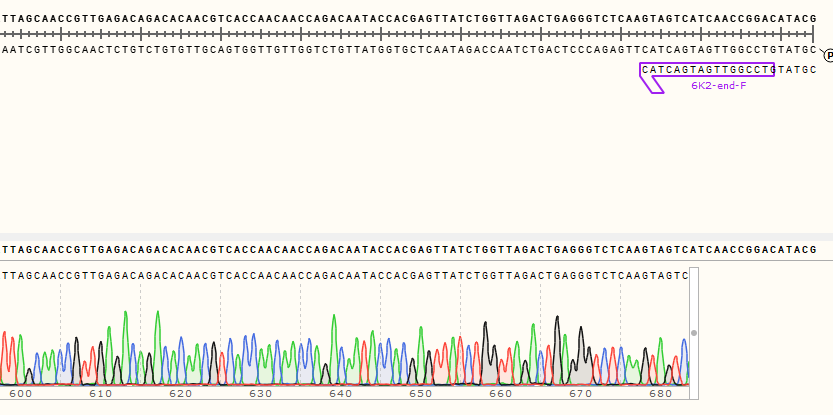

Supplement: Supplementary file 1 [file Table1.docx]
